# A Colourachal-Cutaneous Fistula: A Report of an Unusual Case

**DOI:** 10.1155/2013/865852

**Published:** 2013-01-31

**Authors:** Mohamed Mourad Gargouri, Rami Boulma, Ahmed Sallemi, Mohamed Chlif, Zouhaier Fitouri, Yosri Kallel, Yassine Nouira

**Affiliations:** Department of Urology, La Rabta University Hospital, 1007 Tunis, Tunisia

## Abstract

Urachal anomalies are rare affections due to incomplete closure of urachus during foetal period. Colo-urachal-cutaneous fistula is an uncommon complication of urachal anomalies. Only three cases have been reported so far in the literature. Herein, we report a new case in a 51-year-old patient presenting with umbilical feculent discharge lasting for 4 weeks. Diagnosis was made by computed tomography. After en bloc total surgical excision followup was uneventful.

## 1. Introduction

The urachus represents the vestigial remnant of the allantoids which in the foetus communicates with the cloacae. It normally obliterates by the fifth week of gestation, becoming the median umbilical ligament. Urachal anomalies are rare. They are due to its incomplete closure, which leads to 4 variations: patent urachus, urachal cyst, urachal-umbilical sinus, and vesicourachal diverticulum.

Colo-urachal fistula is a very rare disease; only few cases have been reported, so far, in literature. Herein, we present a new case of colo-urachal-cutaneous fistula with an umbilical feculent discharge.

## 2. Case Report

**Figure 1:**
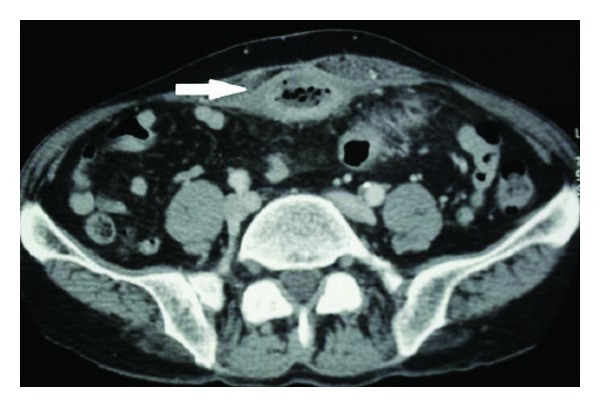
CT showed an air-fluid collection in the urachus (white arrow).

**Figure 2:**
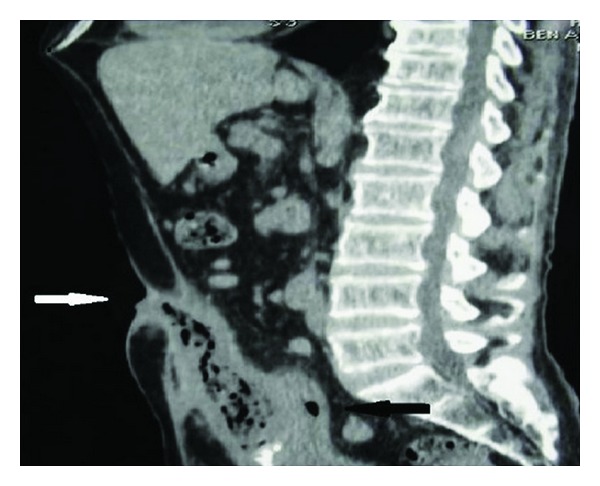
CT reconstruction image revealed a colo-urachal-cutaneous fistula (urachal-cutaneous fistula, white arrow), (colo-urachal communication, black arrow).

A 51-year-old man was hospitalized for feculent umbilical discharge lasting for 4 weeks. He had neither change in bowel transit, nor pneumaturia, nor hematuria, nor dysuria. His past medical history was unremarkable except for femur prosthesis for trauma a long time before. Physical examination revealed a periumbilical erythema surrounding a fistula from the umbilicus, producing feculent liquid. He had normal temperature. The abdomen was soft on examination. Laboratory tests were normal except for white blood cell count of 13.000/mm^3^ and high C-reactive protein. Urinalysis and culture from umbilical fluid were negatives. Colonoscopy showed an abnormal fixation of the left colon to abdominal wall but no area of malignancy was suspected. A computed tomography scans (CT) revealed a 5 cm air-fluid collection extending from the umbilicus to the bladder dome, communicating within the sigmoid colon and the skin from an umbilical-cutaneous fistula (Figures 1 and 2).

**Figure 3:**
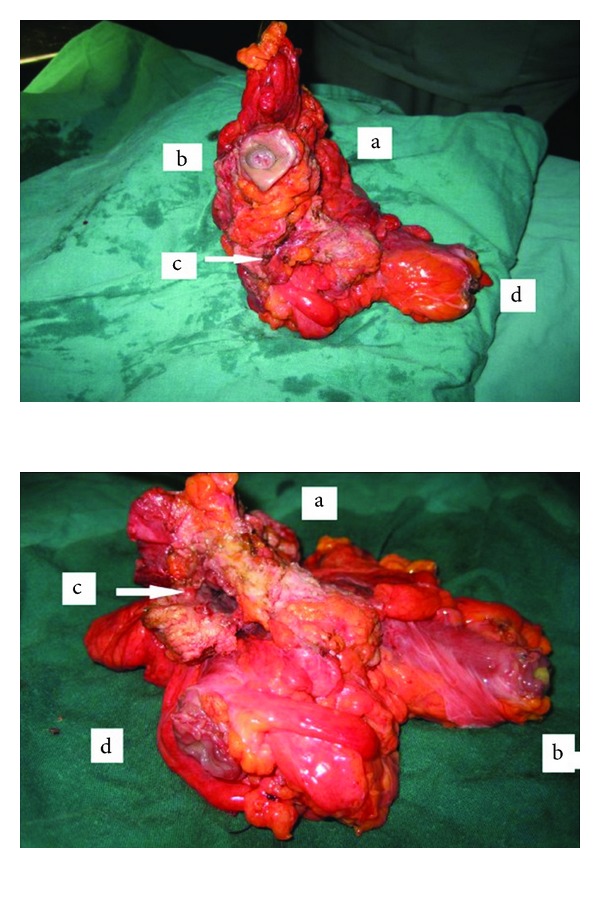
Operative specimen: An en bloc excision of the urachus mass (a), umbilicus (b), a bladder cuff (c), and sigmoid colectomy (d).

The diagnosis of urachal cyst with a sigmoid-urachal-cutaneous fistula was made and surgical treatment was decided. Laparotomy showed an infected urachal cyst, perforated in sigmoid colon with a cutaneous umbilical fistula. An en bloc excision of the urachus mass, umbilicus, a bladder cuff, and sigmoid colectomy with Hartmann's procedure were realized (Figure 3). Cefotaxim and metronidazole antibiotics were started preoperatively and were continued for 3 weeks. Pathological examination showed an inflamed colon with a patent urachal remnant. Colon continuity was established 3 months later and followup was uneventful.

## 3. Discussion

Embryologically, the urachus represents the vestigial remnant of the allantoids which in the foetus communicates with the cloacae. During foetal development, as the bladder descends into the pelvis, its apical portion narrows progressively into a fibromuscular strand, forming the urachus. Normally, it obliterates by the fifth week of gestation, eventually becoming the median umbilical ligament, realising a fibrous cord extending from the dome of the bladder to the umbilicus. It is situated between the transversalis fascia and the peritoneum and is flanked on either side by the lateral umbilical ligaments.

Urachal anomalies are rare and commonly present in early childhood [1, 2]. They are due to incomplete closure of the urachus, which leads to 4 variations: patent urachus (14%), urachal cyst (36%), urachal-umbilical sinus (49%), and vesicourachal diverticulum (1%) [1]. When the entire structure remains open, a patent tube exists between the umbilicus and the dome of the bladder with possibility of urine leakage from the umbilicus; this is called the patent urachus. A cyst is formed when a partial obliteration disturbance appears while both ends close. If the cyst communicates with either the umbilicus or bladder, it will be called respectively a urachal-umbilical sinus and vesicourachal diverticulum.

In most instances these embryologic defects cause little or no trouble as long as they remain free from infection. Urachal cysts are mainly located near the bladder. The presence of secreting epithelium in the cyst may cause accumulation of secretion which leads to an increase in size sufficient to cause symptoms even in the absence of infection [1].

Although there have been several reported cases of enterourachal fistulas involving small intestine mainly as a result of Crohn's disease, a colonic involvement is rare. In fact, only seven cases have been reported, so far, in the literature. The first case was described in 1945 by Sawyer, diagnosing a fistula between the sigmoid colon and a large urachal cyst due to diverticulitis [3]. More recently, six other cases have been published in pubmed indexed journals [4, 5]. From these, 3 sigmoid-urachal-cutaneous fistulas have been reported [4, 6].

The exact number was difficult to determine as keyword terms varied from colourachal, urachal-sigmoid, colourachocutaneous, and sigmoid-urachal-cutaneous fistulas.

The clinical presentation of urachal anomalies is varied; patients can be asymptomatic, describe abdominal pain, irritative voiding symptoms, or umbilical discharge [6]. An umbilical feculent discharge orients toward communication with digestive tube.

Diagnosis of colo-urachal can be defined radiologically; CT reconstruction images can show urachal cyst with communicatin within the colon and umbilicus. Colonoscopy is useful to search for colon malignancy prior to surgery and cystoscopy can be done if an urachal carcinoma is suspected in CT or in case of pneumaturia. Magnetic resonance imaging is a useful modality to evaluate the relationship with surrounding organs and the continuity of urachal cysts to the umbilicus; it could be done if the fistula could not be recognized by CT [2].

Colo-urachal fistulas can be secondary to diverticulitis pathology (4 cases; 50%) which irritates a preexisting urachal cyst [3, 4, 6–8]. For other cases, fistula is probably due to fistulisation into colon of an infected urachal cyst after a long irritation period. Umbilical communication of colo-urachal cyst is either due to a preexisting urachal-umbilical sinus or due to its reopening after postnatal regression. Treatment of urachal cyst is surgical. In a review of the literature, Blichert-Toft and Nielson found that 31% of infected urachal cysts treated by incision and drainage recurred [9]. Since then, treatment of urachal anomalies consists of a complete surgical excision of the urachus including the umbilicus and a bladder cuff to avoid recurrence or development of carcinoma in unresected tissue.

Treatment of colo-urachal fistula consists of en bloc excision of the urachus remnant and colon segmental resection. Antibiotics and percutaneous drainage are commonly performed prior to surgery [4, 6]. Colic anastomosis can be established primary or delayed according to per operative conditions.

## 4. Conclusion

Colo-urachal-cutaneous fistula is a rare condition especially in adulthood. Patients could present with umbilical feculent discharge. The diagnosis can be defined radiologically with CT scans. The treatment consists of complete surgical excision of the urachus including the umbilicus, a bladder cuff, and the pathological colon.
